# *Asplenium merapohense* (Aspleniaceae), a new species from the Peninsular Malaysia

**DOI:** 10.3897/phytokeys.89.20875

**Published:** 2017-11-03

**Authors:** Razali Jaman, Imin Kamin, Ruth Kiew

**Affiliations:** 1 11, Jalan 2/3B, 43650 Bandar Baru Bangi, Selangor, Malaysia; 2 Forest Research Institute Malaysia, 52109 Kepong, Selangor, Malaysia

**Keywords:** *Asplenium*, limestone, flora, morphology, taxonomy

## Abstract

A new species of *Asplenium* is described from two collections made on limestone hills in Peninsular Malaysia. Conspicuous by its extremely narrow pinnae, it is probably allied to *A. salignum* but differs in sufficient characters (scale size, size and shape of lamina, venation and sorus length, position and orientation) to be a species in its own right.

## Introduction

Field work for the Flora of Peninsular Malaysia project focuses on regions that are poorly known botanically (Kiew and Rafidah 2007). This striking new species was discovered during a botanical survey of the limestone karst hills in the Merapoh region, Pahang, Peninsular Malaysia. The outstanding character of this new species is its extremely narrow linear pinnae with a wavy margin, the result of alternating bulges where the sori are positioned. It is an extremely rare species. Only one localised population was encountered on just one hill out of the 15 hills surveyed. However, a search in the herbarium of the Singapore Botanic Gardens (SING) and the Royal Botanic Gardens, Kew (K) produced one other specimen, also from a karst hill, Gua Panjang, Kelantan, about 25 km north of the original site. None of the limestone hills in this area are legally protected and they are currently threatened by quarrying for cement and burning associated with agricultural activities that encroach right up to the foot of the karsts. Due to its rarity and imminent threat, this new species is clearly endangered.

Parris and Latiff (1997) recorded 52 named taxa of *Asplenium* from Malaysia of which 26 occur in Peninsular Malaysia. The most recent comprehensive account for Peninsular Malaysian species still remains that of Holttum (1968). This new species with its short, creeping rhizome with brown-hairy roots and a tuft of simple or once branched light green fronds, pinnae with rounded marginal teeth, 10–20 cm long, subequal at the base, with a broad and raised midrib on the upper surface, and broad, pale indusia most closely resembles *Asplenium salignum* Blume but this new species is different in the combination of characters given in Table 1. Holttum (1968) suggested that in *A. salignum* pinnae width and thickness often depended on degree of exposure, which accounts for some of the variability in this species. This is not the case for the new species because, even after three years’ cultivation in optimum nursery conditions, the pinnae remain as small and as narrow as they were in the wild.

**Table 1.** Differences between *Asplenium merapohense* and *A. salignum*.

Character	*A. merapohense* sp. nov.	*A. salignum* Blume
Scale size (mm)	2–3 × 0.5–0.8	6–10 × 1.4–1.7
Pinna shape	narrowly lanceolate to almost linear	narrowly elliptic
Pinna size (cm)	3.4–7.1 × 0.2–0.3(–0.45)	6–27 × 0.9–3.7
Pinna base	gradually attenuate	cuneate
Secondary veins	unbranched	usually forked near midrib
Sori length (mm)	2–6	4–13
Sori position	on vein in widest part of pinna	along acroscopic vein branches
Sori direction	parallel to midrib	45° to midrib

The extremely narrow pinnae, the striking and characteristic feature of this species, are not seen in Thai species of *Asplenium* (Tagawa and Iwatsuki 1979). An extraordinary *Asplenium*, *A. septentrionale* (L.) Hoffm., widespread from N America to Asia, has even narrower grass-like fronds 1–2.5 mm wide but the rachis is forked towards the apex so the frond is neither simple nor pinnate. It is different too in its venation and its sori that at maturity cover the entire frond surface (Lin and Viane (2013) 272, figure 408.9-17).

## Taxonomy

### Asplenium merapohense

Taxon classificationPlantaePolypodialesAspleniaceae

R.Jaman & K.Imin sp. nov.

urn:lsid:ipni.org:names:77167027-1

Figure 1

#### Diagnosis.

Similar to *Asplenium salignum* in its short creeping rhizome, tuft of simple or once pinnate fronds dentate with rounded marginal teeth but *A. merapohense* is distinct in its shorter, much narrower pinnae 3.4–7.1 × 0.2–0.3(–0.45) cm (vs. 6–27 × 0.9–3.7 cm) and shorter sori, which are 2–6 mm long and lie parallel to the midrib (vs. sori 4–13 mm long angled at 45° to the midrib).

#### Type.

Peninsular Malaysia. Pahang, Lipis District, Merapoh, opposite to Gua Gajah, 4°42.26'N, 101°58.46'E at 348 m, 28 May 2014, Imin et al. FRI 81479 (holotype KEP!; isotypes K!, SAN!, SING!, UKMB!).

#### Description.

Lithophyte. **Rhizome** short to 2 cm long, shortly creeping, slender (to 3 mm diam.), rhizome apex with narrowly triangular clathrate scales, 2–3 × 0.5–0.8 mm, dark brown with slightly paler margin with a few protuberances. **Fronds** tufted and quite close together, once pinnate with 1–2 pairs of pinnae or occasionally the lower pinna unequally forked; stipes 2.5–8.5 cm long, pale to mid-green when dry, grooved above, glabrous or with sparse stellate scales when young; rachis 0.8–4.3 cm long, grooved above, glabrous, slightly winged at the junction of the rachis and pinnules; lamina leathery, lower lamina surface paler than the upper surface, lower pinnae opposite, the upper pinnae (if present) alternate or only a single upper pinna present, terminal pinna conform to lateral pinnae; pinnae narrowly lanceolate to almost linear, 3.4–7.1 × 0.2–0.3(–0.45) cm, margin dentate, teeth minutely rounded giving the pinna a scalloped-edge look, base of pinnae gradually attenuate, midrib raised on upper surface and slightly raised below, apex attenuate; veins alternate, almost invisible, oblique to midrib, free and unbranched, terminating slightly below the lobe/tooth. **Sori** elongate and embedded on acroscopic veins, 2–6 mm long, indusium c. 1 mm wide, pale brown and opening towards the midrib of the pinna. **Spores** ellipsoid, 45–52 × 32–37 µm.

**Figure 1.**
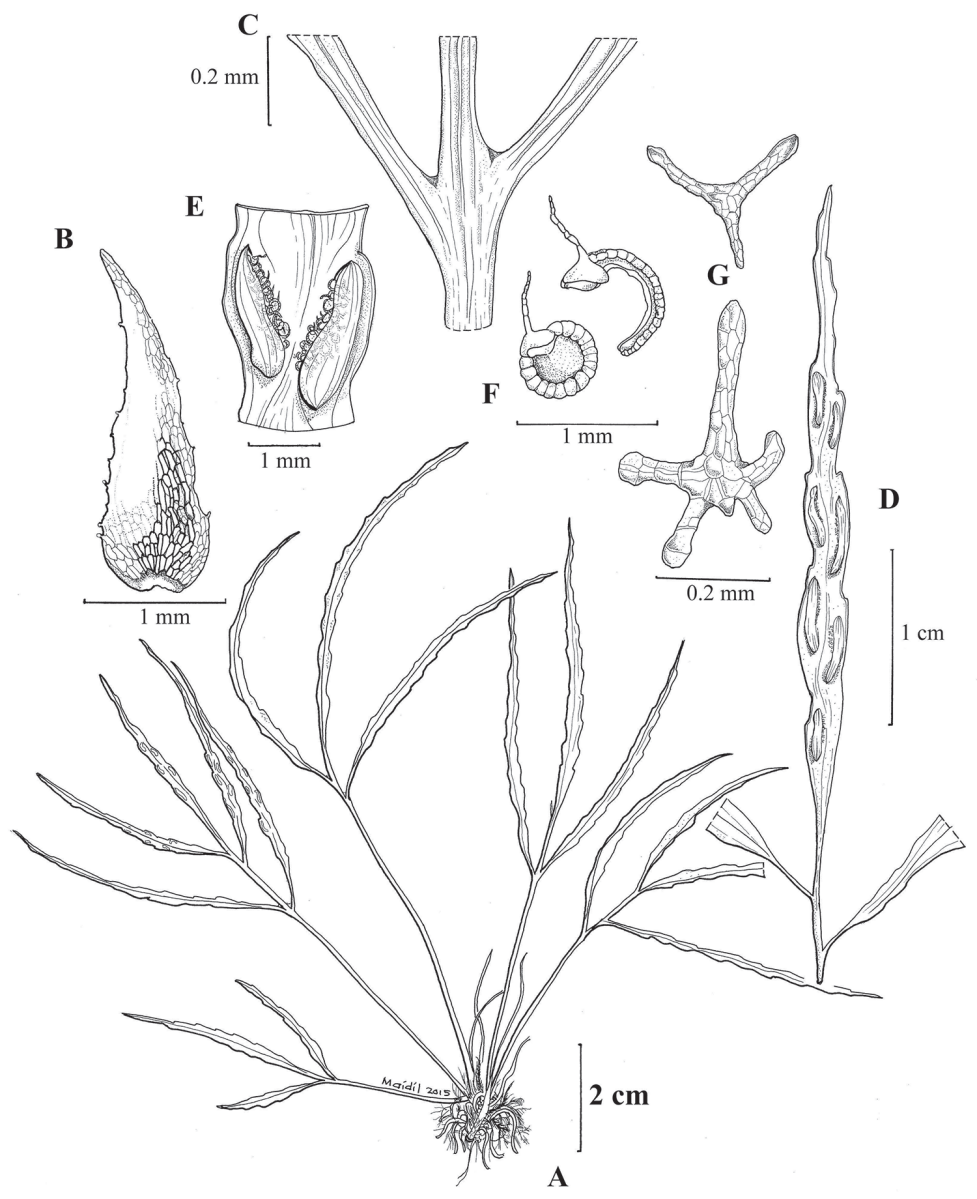
*Asplenium merapohense* R.Jaman & K.Imin, sp. nov. **A** habit **B** rhizome scale **C** rachis with slightly winged axils **D** pinna with sori **E** sori with sporangia **F** sporangia before and after dehiscence **G** 3- and 5-armed scales on lower pinna surface. (Drawn by Mohamad Aidil Noordin from Imin et al. FRI 81479).

#### Distribution.

Endemic in Peninsular Malaysia, from Pahang, Merapoh (Gua Gajah) and Kelantan, Gua Musang District (Gua Panjang).

#### Etymology.

It takes its name from the type locality.

#### Conservation status.

Critically Endangered, CR B2ab(iii). Known from only two localities with an AOO of 4 km^2^ (not far apart, so the two sites count as a single location), its small population (the population on Gua Gajah comprises about 100 individuals; the Gua Panjang site has not been relocated), and the threats to its habitat from quarrying for cement and burning for agricultural purposes, all contribute towards its critically endangered status. A few fertile plants are growing in the Herbarium Nursery at the Forest Research Institute Malaysia.

#### Ecology.

**Figure 2.**
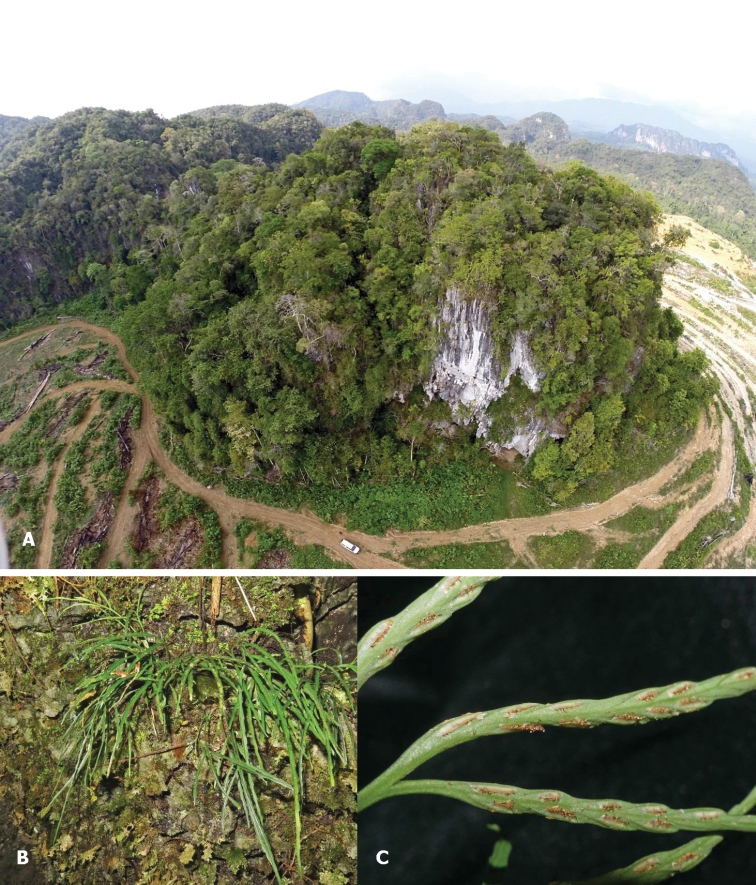
*Asplenium merapohense* R.Jaman & K.Imin, sp. nov. **A** Gunung Gajah **B** habitat – in crevices on a shaded, mossy limestone vertical cliff **C** Undersurface of a pinna with sori showing indusia. (Photographs by **A** P.T. Ong, **B, C** K. Imin).

Restricted to karst limestone hills where it grows on steep rock faces in sheltered conditions at 180–348 m altitude. It is a rare and very local species (Figure 2).

#### Other specimens examined.

**Peninsular Malaysia.** Pahang: Merapoh, opposite Gua Gajah-28 May 2014 Imin et al. FRI 81479 (KEP!, K!, SAN!, SING!, UKMB!). Kelantan: Gua Panjang-10 Aug 1962 UNESCO Limestone Expedition 586 (K!, SING!).

#### Discussion.

*Asplenium salignum* is a widespread species in Peninsular Malaysia, most commonly encountered on limestone rocks but also on granite rocks in lowland forest and occasionally as an epiphyte in mountain forest. It is variable in the division of the fronds: it is sometimes found with only simple fronds, most commonly plants with simple fronds also bear fronds with one or two pairs of pinnae, while many plants have 1–2(–6) pairs of pinnae. However, even small fronds with simple or a single pair of pinnae have wider lateral pinnae (7–) 14–20(–28) mm wide with wider terminal pinnae (11–)15–25(–46) mm wide compared to the new species that has pinnae 2–3(–4.5) mm wide. Another conspicuous difference is in the size and position of the sori. In *A. merapohense* the sori are 2–6 mm long and lie parallel to the midrib, while in *A. salignum* they are 4–13 mm long and positioned at 45° to the midrib *Asplenium merapohense* is restricted to limestone, but while *A. salignum* is usually found on limestone, it also grows as an epiphyte in mountain forest.

The fact that this new species is extremely rare and known from a single population might suggest that it is a hybrid. Murakami et al. (1991) recorded that several non-fertile natural hybrids of *Asplenium* occur in Japan and that they were frequent where both parents occurred.

The hybrid status of apparently fertile hybrids was revealed by their misshapen spores. On the karst limestone in the Merapoh region, four other *Asplenium* species occur, *A. batuense* Alderw., *A. macrophyllum* Sw., *A. normale* Don and *A. salignum*, but *A. merapohense* does not display any characters intermediate between them and *A. salignum*, the most likely putative parent if it were a hybrid. In addition, examination of the spores of *A. merapohense* show them to be of regular size and shape, the fronds are not malformed, and the population numbers about 100 or more individuals in all life stages, indicating that it is fully fertile, all of which strongly indicate that it is not a hybrid.

## Supplementary Material

XML Treatment for Asplenium merapohense
